# Supplementary material for: Cell type specific transcriptional reprogramming of maize leaves during Ustilago maydis induced tumor formation
Source: Sci Rep. 2019 Jul 15;9:10227. doi: 10.1038/s41598-019-46734-3 (PMC6629649; doi:10.1038/s41598-019-46734-3)
Supplement: Supplementary file 1 — Supplementary Figures 1-4 [file 41598_2019_46734_MOESM1_ESM.pdf]

Cell type specific transcriptional reprogramming of maize leaves during *Ustilago maydis* induced tumor formation

Mitzi Villajuana-Bonequi[1], Alexandra Matei[1], Corinna Ernst[2], Asis Hallab[3], Björn Usadel[3] and Gunther Doehlemann[1,*]

[1.]Botanical Institute and Cluster of Excellence on Plant Sciences (CEPLAS), BioCenter, University of Cologne, Zülpicher Str. 47a, Cologne 50674, Germany.

[2.]Center for Familial Breast and Ovarian Cancer, Medical Faculty, University Hospital Cologne, University of Cologne, Cologne 50931, Germany.

[3.]BioSC, IBG-2, Institute of Botany, RWTH Aachen, Worringer Weg 3, Aachen 52074, Germany

[*]To whom correspondence should be addressed:
g.doehlemann@uni-koeln.de

Tel: +49 221-470-1647
Fax: +49 221-470-7406

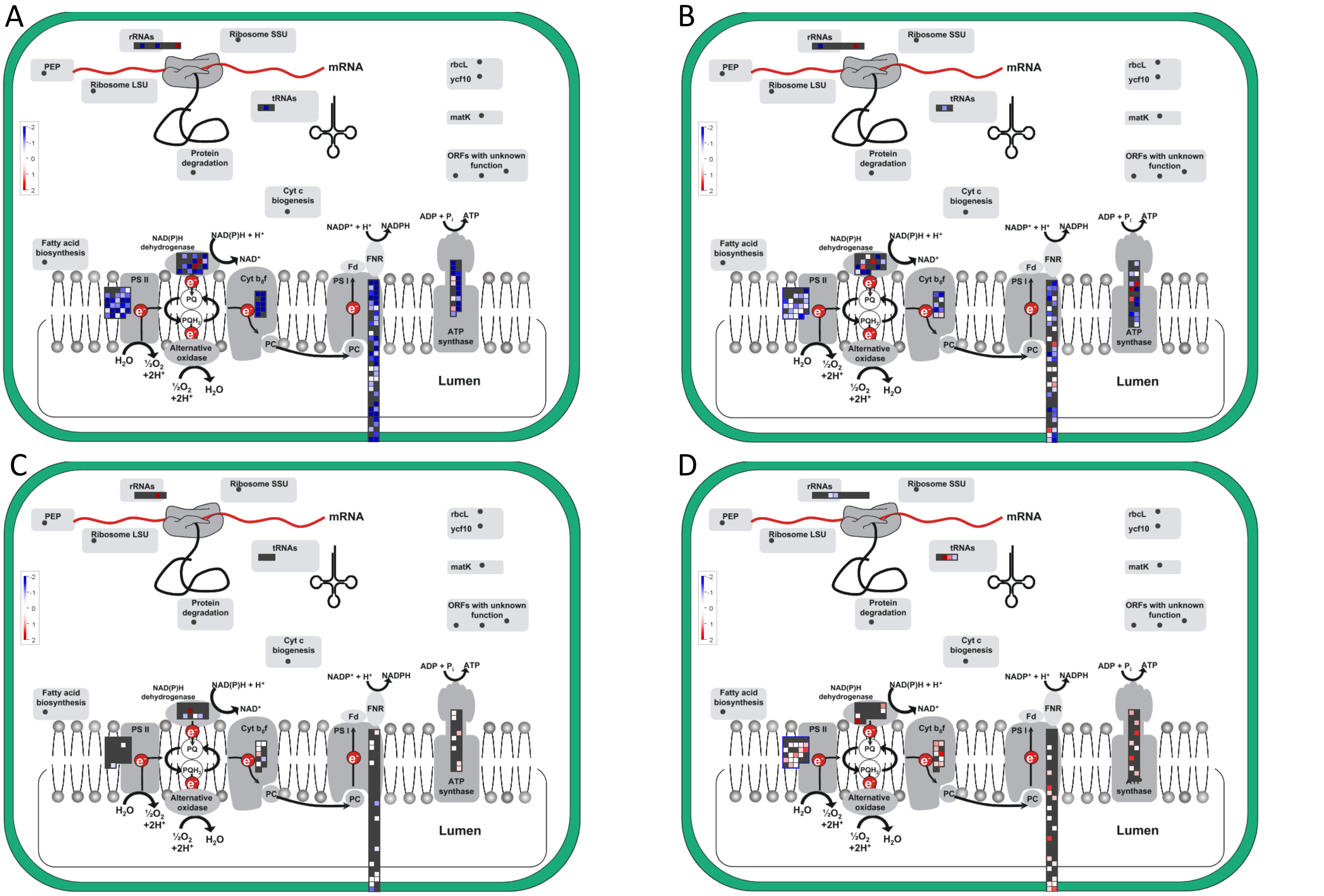

Supplementary Figure 1. Chloroplast responses to *Ustilago maydis* infection in specific cell-types. Genes differentially expressed (FDR ≤5%) are shown A, HPT. B HTT. C seeTC. D seeTC.vs.HTT: Upregulated transcripts are shown in red and downregulated transcripts are colored blue.

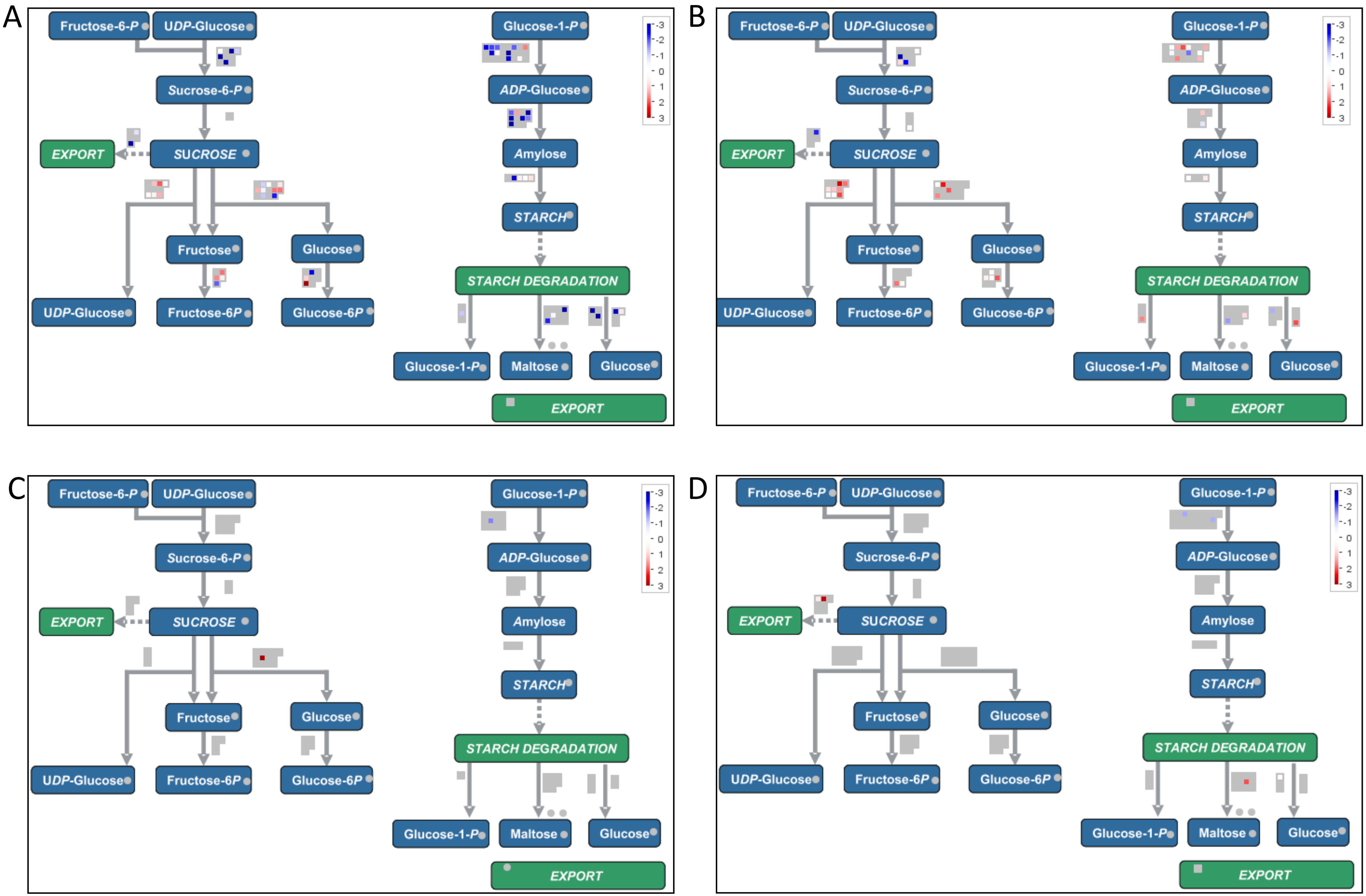

Supplementary Figure 2. Sucrose- and Starch- biosynthesis responses to *Ustilago maydis* infection in specific cell-types. Genes differentially expressed (FDR ≤5%) are shown A, HPT. B HTT. C seeTC. D seeTC.vs.HTT: Upregulated transcripts are shown in red and downregulated transcripts are colored blue.

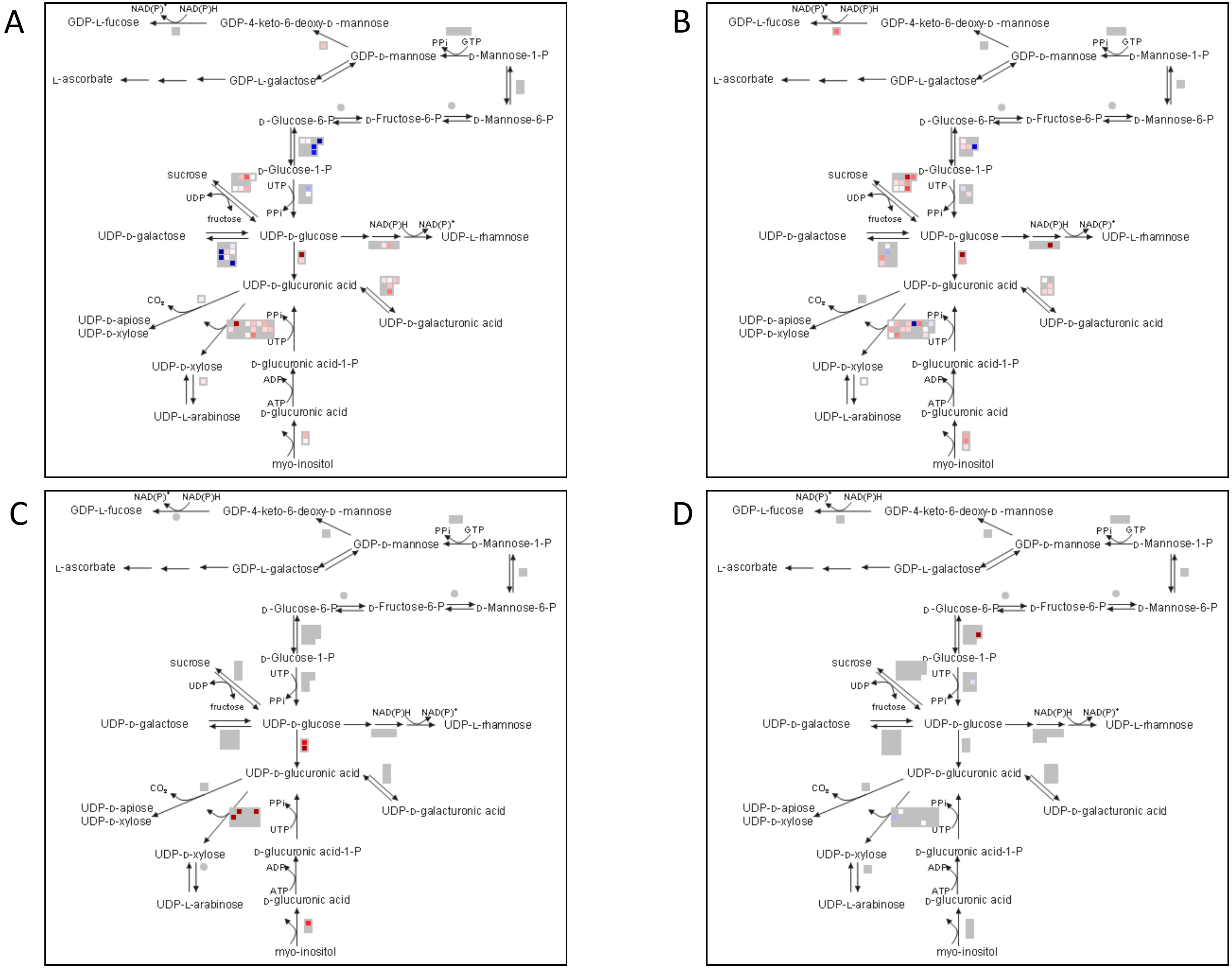

Supplementary Figure 3. Cell wall precursors biosynthesis responses to *Ustilago maydis* infection in specific cell-types. Genes differentially expressed (FDR ≤5%) are shown A, HPT. B HTT. C seeTC. D seeTC.vs.HTT: Upregulated transcripts are shown in red and downregulated transcripts are colored blue.

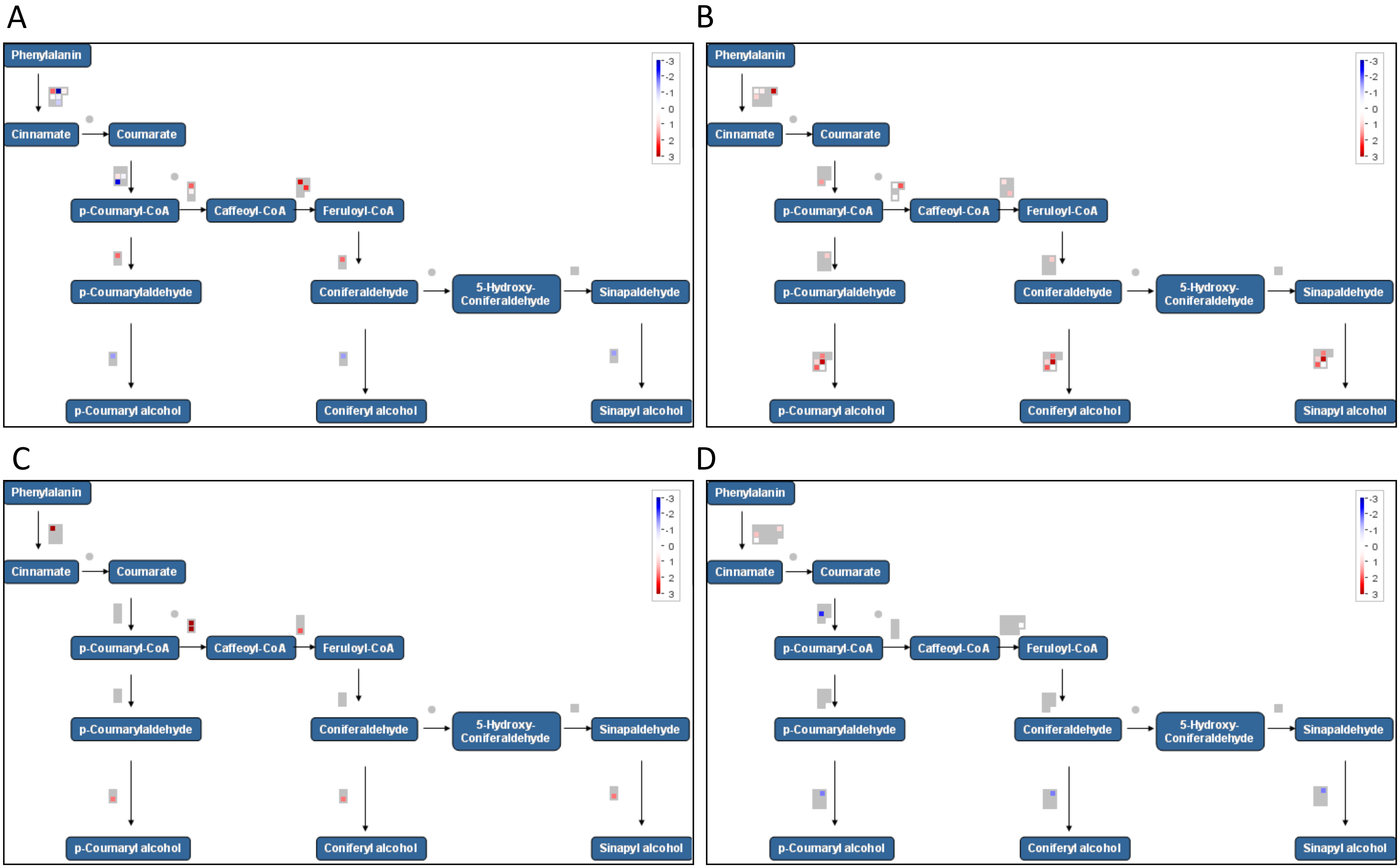

Supplementary Figure 4. Lignin biosynthesis responses to *Ustilago maydis* infection in specific cell-types. Genes differentially expressed (FDR ≤5%) are shown A, HPT. B HTT. C seeTC. D seeTC.vs.HTT: Upregulated transcripts are shown in red and downregulated transcripts are colored blue.
